# Efficacy and safety of ^177^Lu‑DOTATATE in patients with advanced pancreatic neuroendocrine tumours: data from the NETTER-R international, retrospective study

**DOI:** 10.1007/s00259-022-05771-3

**Published:** 2022-04-07

**Authors:** Dominique Clement, Shaunak Navalkissoor, Rajaventhan Srirajaskanthan, Frédéric Courbon, Lawrence Dierickx, Amy Eccles, Valerie Lewington, Mercedes Mitjavila, Juan Carlos Percovich, Benoît Lequoy, Beilei He, Ilya Folitar, John Ramage

**Affiliations:** 1 King’s College Hospital, London, UK; 2 Royal Free Hospital, London, UK; 3 Institut Universitaire du Cancer de Toulouse Oncopole, Toulouse, France; 4 Guy’s and St Thomas’ Hospital, London, UK; 5 Hospital Universitario Puerta de Hierro Majadahonda, Madrid, Spain; 6 Hospital General Universitario Gregorio Marañón, Madrid, Spain; 7 Novartis Pharma AG, Basel, Switzerland

**Keywords:** NET, GEP-NET, panNET, ^177^Lu-DOTATATE, NETTER-R

## Abstract

**Purpose:**

NETTER-R aimed to determine the efficacy, safety and tolerability of ^177^Lu-DOTATATE in patients with progressive, advanced pancreatic neuroendocrine tumours (panNETs) using retrospective real-world data from multiple sites.

**Methods:**

This international study retrospectively included patients with panNETs treated with ^177^Lu-DOTATATE. The primary endpoint was progression-free survival (PFS) by Response Evaluation Criteria in Solid Tumors version 1.1 (RECIST v1.1). Secondary endpoints included overall survival (OS), safety and tumour response.

**Results:**

In total, 110 patients with panNETs were studied; 65.5% received a cumulative dose of ^177^Lu-DOTATATE 29.6 GBq ± 10% (median: 7.4 GBq). In 62 patients with available RECIST v1.1 tumour response, the median PFS was 24.8 months (95% confidence interval [CI]: 17.5–34.5), and the objective response rate was 40.3% (95% CI: 28.1–53.6); all responses were partial. With a median follow up of 24.5 months (range: 2.0–123.4 months) after the first cycle of ^177^Lu-DOTATATE, the median OS in the full analysis set (*n* = 110) was 41.4 months (95% CI: 28.6–50.2). PFS (hazard ratio [HR]: 3.672; *p* = 0.0009) and OS (HR: 3.360; *p* < 0.0001) were longer in patients who received no chemotherapy prior to ^177^Lu-DOTATATE than those who did. No treatment-emergent adverse events (TEAEs) led to treatment discontinuation. Grade 3 anaemia, lymphopenia and thrombocytopenia occurred in 0.9%, 5.4% and 0.9% of patients, respectively. No acute leukaemia or myelodysplastic syndrome was reported. Six patients (5.5%) had renal TEAEs. All renal grade ≥ 3 events were transient and did not lead to treatment modification.

**Conclusions:**

These results reinforce the role of ^177^Lu-DOTATATE for the treatment of patients with advanced, somatostatin receptor-positive panNETs.

**Supplementary Information:**

The online version contains supplementary material available at 10.1007/s00259-022-05771-3.

## Introduction

Gastroenteropancreatic neuroendocrine tumours (GEP-NETs) appear throughout the gastrointestinal (GI) tract, including the pancreas [1], and account for 65–75% of all neuroendocrine tumours (NETs) [2]. GEP-NETs occurring in the pancreas are referred to as pancreatic neuroendocrine tumours (panNETs) and classified as functioning or non-functioning [3].

Although panNETs are rare, their incidence has increased 2- to 10-fold in recent decades [4, 5]. In the USA, the incidence of panNETs is approximately 1 per 100,000 population [6, 7]. In Europe, reported figures range from 0.1 to 0.5 per 100,000 population [8]. Patients with panNETs demonstrate a lower median survival time and survival rate than those with NETs located elsewhere in the GI tract [9].

Several therapeutic strategies for advanced SSTR-positive panNETs exist, including peptide receptor radionuclide therapy with radiolabelled SSAs such as ^177^Lu-DOTATATE [10], targeted treatments (sunitinib and everolimus), as well as chemotherapy and SSAs [10–12]

^177^Lu-DOTATATE belongs to the class of agents known as radioligand treatments. It is approved for the treatment of SSTR-positive GEP-NETs in adults [13, 14], based on data from the NETTER-1 phase III and Erasmus Medical Centre (MC) retrospective cohort trials [10, 15]. The approved treatment regimen of ^177^Lu-DOTATATE consists of four cycles of 7.4 GBq each, every 8 weeks [13, 14]. Current guidelines for the treatment of panNETs recommend the use of PRRT such as ^177^Lu-DOTATATE at second or third line (depending on tumour grade) after progression on other agents [10, 15, 16].

The efficacy and safety of ^177^Lu-DOTATATE in patients with GEP-NETs (including panNETs) have been demonstrated in clinical studies [17–19]. In the phase III NETTER-1 study in midgut NET patients, treatment with ^177^Lu-DOTATATE achieved a clinically and statistically significant improvement in PFS (hazard ratio [HR]: 0.18, 95% confidence interval [CI]: 0.11–0.29; *p* < 0.0001) compared with high-dose octreotide LAR [13]. In the final analysis, with a median follow-up of 6.3 years, treatment with ^177^Lu-DOTATATE led to a clinically relevant improvement in median OS of 11.7 months compared with high-dose octreotide LAR, but the difference did not reach statistical significance (HR: 0.84, 95% CI: 0.60–1.17; *p* = 0.30) [19]. Restricted mean survival time (RMST) was analysed at 2, 3, 4, and 5 years post-randomisation to account for the presence of nonproportional hazards. With a median follow-up of 6.3 years, RMST was numerically longer in the [^177^Lu]Lu-DOTA-TATE arm versus control arm at all time points [19].

The retrospective cohort Erasmus MC study enrolled 1,214 patients with SSTR-positive tumours, including 133 patients with panNETs, and concluded that treatment with ^177^Lu-DOTATATE was efficacious and well tolerated [18]. For patients with panNETs, median PFS, time to progression (TTP), and OS were 30 months, 31 months, and 71 months, respectively. Radiologic disease control was observed in 81% of patients with panNETs. Complete response (CR), partial response (PR), stable disease (SD), and progressive disease (PD) were observed in 6 (5%), 66 (50%), 40 (30%), and 17 (13%) patients [18]. It should be noted that the efficacy analysis was not analysed in an intent to treat manner.

The NETTER-R study reported here builds on previous evidence from the NETTER-1 [17] and Erasmus MC [18] studies to support the use of ^177^Lu-DOTATATE for the treatment of patients with panNETs. It aimed to determine the efficacy, safety and tolerability of ^177^Lu-DOTATATE in patients with progressive, advanced panNETs based on retrospective real-world data from multiple sites.

## Materials and methods

The NETTER-R study was an international, retrospective study of patients treated with ^177^Lu-DOTATATE as per the EU label in the UK, France and Spain, collecting and analysing subjects’ paper-based and electronic medical records.

Participants were identified by investigators or through an early access programme. Approval from the institutional review board and independent ethics committee was obtained before procuring written informed consent for the study, as required by local regulations.

The NETTER-R study was conducted in patients with unresectable or metastatic (based on histology), well-differentiated, SSTR-positive, progressive panNETs (grades 1 and 2) who were treated with ^177^Lu-DOTATATE. Subjects with NETs of other or unknown origins, including those with pancreas involvement and tumours with mixed histology, were excluded from the study. Progressive disease was assessed both radiologically and clinically through investigator opinion. The full planned treatment for each patient consisted of a total cumulative administered radioactivity of 29.6 GBq with the dosing equally divided among 4 administrations of ^177^Lu-DOTATATE (7.4 GBq) at 8 ± 1 week intervals, extendible up to 16 weeks to accommodate resolving acute toxicity.

Safety parameters were collected when available. Routine biological tests recorded in the database included haematological and metabolic evaluations as well as ECG and vital sign monitoring. All available data and routine biological tests related to the study endpoints were collected retrospectively from the medical records of eligible subjects from the baseline visit (prior to receiving the first ^177^Lu-DOTATATE treatment), during ^177^Lu-DOTATATE treatment and then during follow-up visits until the most recent contact available or end of the study. Evaluations had no mandated frequency, and intervals between evaluation visits varied between patients as they were conducted according to each institution’s schedule and assessment protocols. Follow-up data were tentatively collected on a quarterly basis, depending on standard care, local practice, and availability of source documents at sites. Patient data were uploaded onto a pseudonymised electronic case report form (eCRF) via a web-based platform.

The primary endpoint was PFS based on local radiological assessment. In as many cases as possible, the radiological response according to Response Evaluation Criteria in Solid Tumors version 1.1 (RECIST v1.1) was obtained for each patient.

Secondary endpoints included OS, objective response rate (ORR), duration of response (DoR), TTP and PFS as determined by available tumour assessments (including RECIST v1.1, radiological, biochemical, metabolic and clinical assessments). The incidence of treatment-emergent adverse events (TEAEs) was also a secondary endpoint of the study. TEAEs were defined as any adverse event (AE) starting after or on the day of administration with ^177^Lu-DOTATATE. AEs were defined as any untoward medical occurrence in a patient who has been administered ^177^Lu-DOTATATE. All AEs were reported through eCRFs.

Two analysis sets were defined for efficacy and safety evaluations. The full analysis set (FAS) consisted of all subjects who received at least one cycle of ^177^Lu‑DOTATATE and provided data for at least one efficacy endpoint, including OS. All efficacy analyses were primarily performed on the FAS. The safety analysis set (SAS) consisted of all subjects who received at least one cycle of ^177^Lu‑DOTATATE. All safety analyses were performed on the SAS.

Due to the retrospective nature of this study, no formal statistical sample size calculation was performed. The planned sample size of 120 participants was selected to provide sufficient data to reliably estimate efficacy and safety endpoints in this patient population.

Subgroup analyses of PFS and OS were performed using Cox models and two-sided log-rank tests comparing groups with or without prior chemotherapy, targeted agents (protein kinase inhibitors) and SSAs. Time from panNET diagnosis to ^177^Lu-DOTATATE treatment, NET grade and patient age were included in the Cox model. HR was expressed as ‘with prior treatment/without prior treatment’ estimated from the corresponding Cox model. In addition, analysis by the number of prior anticancer treatments was conducted; however, as it was not included in the original statistical analysis plan, significance could not be calculated.

Due to the methodological limitations of the study, the primary focus of the statistical analysis was descriptive statistics and graphical presentations of data. Tumour response evaluation reflects real-world assessment practices and include locally reviewed RECIST v1.1 evaluation as well as other response assessments by the local investigator, such as clinical or biological data. To categorise these assessments, three assessment groups were derived:

1. RECIST v1.1 tumour assessments (*n* = 62).
2. Investigator opinion 1: In addition to RECIST v1.1, radiological assessments (different from RECIST v1.1) were used if RECIST v1.1 assessments were not available (*n* = 83).
3. Investigator opinion 2: Any assessment was used (RECIST v1.1, radiological, clinical, biomarker or metabolic assessment), by order of availability (*n* = 100).

PFS, TTP, ORR and DoR were reported for each of the three categories above.

## Results

### Patient characteristics and treatment exposure

A total of 110 patients with panNETs were identified (UK, *n* = 66; France, *n* = 21; Spain, *n* = 23). At the start of treatment, the median age was 58.0 years (range: 28.0–89.0 years), the median body weight was 68.0 kg (range: 42.0–138.0 kg), and 47.3% were female. At baseline, 96.4% of patients had progressive disease. The median time since first diagnosis was 42.6 months, with a median of 38.1 months since first diagnosis of metastasis. Most patients had WHO NET grade 2 tumours and evidence of liver metastases. A total of 9 (8.2%) patients received off-label treatment, 3 (2.7%) of whom had grade 3 tumours and 6 (5.5%) had missing information. These patients were included in the study as they had already received treatment with ^177^Lu-DOTATATE from their local institution. In total, 91.8% of patients had received prior anticancer therapy (Table 1 and Supplementary Table 1).

**Table 1:** Baseline patient characteristics (SAS, *n* = 110)

**Patient demographics**	
Age (years)	
Q1–Q3 Median Min–max	50.0–66.0 58.0 28–89
Weight (kg)	
Q1–Q3 Median Min–max	58.7–77.8 68.0 42.0–138.0
Sex, *n* (%)	
Female Male	52 (47.3) 58 (52.7)
**Tumour evaluation**	***n (%)***
WHO NET grade	
NET, G1	30 (27.3)
NET, G2	71 (64.5)
NET, G3	3 (2.7)
Missing	6 (5.5)
Site of metastasis	
Liver	105 (95.5)
Lymph nodes	47 (42.7)
Bone	32 (29.1)
Lungs	4 (3.6)
Tumour status	
Functional	33 (30.0)
Non-functional	63 (57.3)
Not assessed	12 (10.9)
Missing	2 (1.8)
**Treatment history**	***n (%)***
Patients having previously received anticancer therapy	
Yes	101 (91.8)
No	9 (8.2)
Progression at baseline^a^	
Yes	106 (96.4)
No	4 (3.6)

^a^Patients without progression at baseline were diagnosed with metastatic panNETs within 3 months of receiving the first cycle of ^177^Lu-DOTATATE

*G1*, grade 1; *G2*, grade 2; *G3*, grade 3; *max*, maximum; *min*, minimum; *NET*, neuroendocrine tumour; *panNET*, pancreatic neuroendocrine tumour; *Q*, quartile; *SAS*, safety analysis set; *WHO*, World Health Organization

Most patients (70.0%) received all four scheduled cycles of ^177^Lu-DOTATATE (one cycle, 6.4%; two cycles, 11.8%; three cycles, 10.9%; five cycles, 0.9%). Some patients stopped treatment early due to progressive disease (10%), death (6.4%), adverse events (0.9%), or other reasons (6.4%). The median interval of time between each treatment cycle was 10.6 weeks. The cumulative activity was 29.6 GBq ± 10% (26.6–32.6 GBq) in 65.5% of patients (< 26.6 GBq: 31.8%, ≥ 32.6 GBq: 2.7%). Twelve patients received 1–4 additional cycles of ^177^Lu-DOTATATE after the initial treatment (Table 2 and Supplementary Table 2).

**Table 2:** PRRT treatment characteristics

	Initial treatment period, *n* = 110	Additional treatment period, *n* = 12
Cumulative activity (GBq), *n* (%)		
< 26.6	35 (31.8)	
26.6– < 32.6	72 (65.5)	
≥ 32.6	3 (2.7)	
Number of cycles		
Median	4.0	2.0
Min–max	1–5	1–4
Average activity per cycle (GBq)		
Median	7.4	7.4
Min–max	3.7–8.3	3.7–7.9
Average duration between treatment cycles per patient (weeks)		
Median	10.6	
Min–max	8.4–15.0	

*max*, maximum; *min*, minimum; *PRRT*, peptide receptor radionuclide therapy

The median follow-up after the first cycle of ^177^Lu-DOTATATE was 24.5 months (range: 2.0–123.4 months).

### Efficacy

Of the 110 enrolled patients, tumour response assessment per RECIST v1.1 (locally reviewed) was available for 62 patients but was not available in 48 patients, which is common for retrospective real-world studies. Tumour response data according to investigator opinion 1 were available for 83 patients, and 100 patients had at least one tumour assessment performed after baseline (assessable according to investigator opinion 2). The differences between the patients for whom RECIST v1.1 evalution data were available (56.4%) were compared with those for whom RECIST v1.1 data were not available (43.6%). The groups with and without RECIST v1.1 data were clinically comparable in terms of demography, histopathological profile and time from diagnosis.

**Fig. 1:**
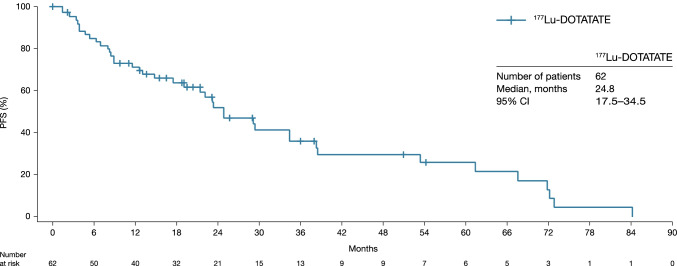
Kaplan–Meier analysis of PFS by RECIST v1.1. This analysis includes patients in the FAS where RECIST v1.1 data were available (*n* = 62).* CI*, confidence interval; *FAS*, full analysis set; *PFS*, progression-free survival; *RECIST v1.1*, Response Evaluation Criteria in Solid Tumors version 1.1

**Fig. 2:**
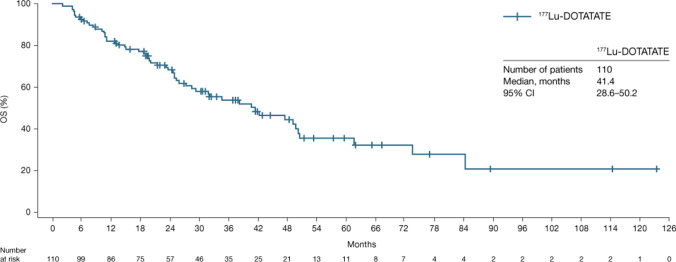
Kaplan–Meier analysis of OS. This analysis includes patients in the FAS where OS data were available (*n* = 110). *CI*, confidence interval; *FAS*, full analysis set; *OS*, overall survival

In the 62 patients with tumor response assessed by RECIST v1.1, the median PFS was 24.8 months (95% CI: 17.5–34.5) (Table 3; Fig. 1), the median OS (*n* = 110) was 41.4 months (95% CI: 28.6–50.2) (Fig. 2), the median TTP was 29.5 months (95% CI: 21.4–67.6) (Table 3), the median DoR in the 25 responders was 60.7 months (95% CI: 13.1–62.1) (Table 3), and ORR (PR + CR) was 40.3% (95% CI: 28.1–53.6) (Table 3). The ORR by investigator opinion 2 (*n* = 100) was 54.0% (95% CI: 43.7–64.0), including two patients with CR (Table 3 and Supplementary Table 3). The two cases of CR recorded by investigator opinion 2 were detected by the additional clinical, metabolic and biomarker examinations involved in this type of assessment as no radiological assessments were performed at the time of clinical response. Notably, approximately 30% of patients experienced disease progression (Fig. 1) within the first 9 months of study.

**Table 3:** Median PFS, median TTP, median DoR, and ORR results

	*N*	PFS^a^, months (95% CI)	TTP^b^, months (95% CI)	ORR^c^, *n* (%)	DoR^d^, months (95% CI)
RECIST v1.1	62	24.8 (17.5–34.5)	29.5 (21.4–67.6)	25 (40.3)	60.7 (13.1–62.1)
Investigator opinion 1	83	24.0 (19.8–31.3)	27.9 (21.4–37.2)	36 (43.4)	31.1 (16.8–62.1)
Investigator opinion 2	100	24.0 (19.8–29.7)	29.2 (21.4–32.3)	54 (54.0)	28.3 (16.8–60.7)

^a^Defined as the time from treatment start date to documented locally assessed disease progression or death due to any cause. ^b^Defined as the time from treatment start to tumour progression. ^c^Calculated as the proportion of subjects with PR or CR during the observation period. ^d^Defined as the time from initially meeting the criteria for response (CR or PR) until the time of disease progression (PD)

Ten patients were not included in the analysis due to lack of progression at baseline or tumour assessment

Investigator opinion 1 = RECIST v1.1 tumour assessments and radiological assessments. Investigator opinion 2 = all assessments available (radiological, clinical, metabolic and biomarker assessments)

*CI*, confidence interval; *CR*, complete response; *DoR*, duration of response; *ORR*, objective response rate; *PD*, progressive disease; *PFS*, progression-free survival; *PR*, partial response; *RECIST v1.1*, Response Evaluation Criteria in Solid Tumors version 1.1; *TTP*, time to progression

The subgroup analyses by the type of prior anticancer treatments demonstrated that OS (HR: 3.360, *p* < 0.0001) and PFS by RECIST v1.1 (HR: 3.672, *p* = 0.0009) appear to be longer in patients who had not received prior chemotherapy compared with those that had (Table 4). For patients with/without prior PKIs, there was no difference in PFS by RECIST v1.1. However, longer OS was demonstrated in patients without prior PKIs (HR: 2.187, *p* = 0.0128) than those with prior PKIs. No significant difference in PFS or OS was observed between patients with/without prior SSAs. Further assessment revealed that response to ^177^Lu-DOTATATE appeared less favourable in patients who received more than two prior anticancer therapies than in those who received fewer than two therapies (Supplementary Table 4). However, all subgroup analyses should be interpreted with caution as they were exploratory in nature, did not account for multiplicity and were not included in the original statistical analysis plan.

**Table 4:** PFS and OS by prior treatment type

	PFS, months, by RECIST v1.1		PFS, months, by investigator opinion 1		PFS, months, by investigator opinion 2		OS, months	
With prior chemotherapy	14.9 (*n* = 33)	HR: 3.672 *p* = 0.0009	19.1 (*n* = 40)	HR: 2.642 *p* = 0.0032	17.5 (*n* = 47)	HR: 2.568 *p* = 0.0009	24.8 (*n* = 52)	HR: 3.360, *p* < 0.0001
Without prior chemotherapy	38.3 (*n* = 29)		34.5 (*n* = 43)		32.3 (*n* = 53)		61.5 (*n* = 58)	
With prior PKI^a^	23.5 (*n* = 24)	HR: 1.538 *p* = 0.1615	18.7 (*n* = 32)	HR: 1.748 *p* = 0.0287	12.7 (*n* = 36)	HR: 2.208 *p* = 0.0017	28.6 (*n* = 42)	HR: 2.187, *p* = 0.0128
Without prior PKI	24.8 (*n* = 38)		29.5 (*n* = 51)		29.5 (*n* = 64)		49.2 (*n* = 68)	
With prior SSA^b^	24.8 (*n* = 42)	HR: 1.114 *p* = 0.7923	23.3 (*n* = 57)	HR: 1.322 *p* = 0.6130	23.3 (*n* = 71)	HR: 1.227 *p* = 0.8167	47.5 (*n* = 77)	HR: 1.127, *p* = 0.9414
Without prior SSA	24.8 (*n* = 20)		29.2 (*n* = 26)		29.2 (*n* = 29)		32.2 (*n* = 33)	

^a^PKIs used included everolimus, sunitinib and dactolisib. ^b^Somatostatin and analogues

*p* value is derived from a two-sided log-rank test between groups with or without prior anticancer treatments. Subgroup analysis was done using the Cox model to compare patients between the two groups for PFS and OS. Time from panNET diagnosis to ^177^Lu-DOTATATE treatment, NET grade and patient age are included in the Cox model. HR is expressed as ‘with prior treatment/without prior treatment’ estimated from the corresponding Cox model

*HR*, hazard ratio; *NET*, neuroendocrine tumour; *OS*, overall survival; *panNET*, pancreatic neuroendocrine tumour; *PFS*, progression-free survival; *PKI*, protein kinase inhibitor; *RECIST v1.1*, Response Evaluation Criteria in Solid Tumors version 1.1; *SSA*, somatostatin analogue

### Safety

At least one TEAE occurred in 71.8% (79/110) of patients. The most frequent TEAEs were nausea (31/110, 28.2%), fatigue (25/110, 22.7%) and abdominal pain (18/110, 16.4%), predominantly grade 1/2 in severity. Treatment modification was required in 9.1% (10/110) of patients, and no TEAEs led to treatment discontinuation. Grade 5 TEAEs occurred in 2.7% (3/110) of patients; they were abdominal abscess (1/110, 0.9%), metabolic encephalopathy (1/110, 0.9%) and pulmonary embolism (1/110, 0.9%) (Supplementary Table 5).

Some haematological toxicities were observed. Grade 3 anaemia, lymphopenia and thrombocytopenia occurred in one (0.9%), six (5.4%) and one (0.9%) patients, respectively. No grade ≥ 3 neutropenia was reported (Supplementary Table 6). No secondary haematological malignancies, including acute leukaemia (AL) or myelodysplastic syndrome (MDS), were reported during the treatment or follow up (the median follow-up was 24.5 months [range: 2.0–123.4 months]).

Renal TEAEs occurred in six patients (5.5%), of which three were grade 3. Two patients (1.8%) developed acute kidney injury, including one (0.9%) with grade 3 severity. Renal impairment was also reported in two patients (1.8%), including one (0.9%) with grade 3 severity. Haematuria and renal failure were each observed in one patient (0.9%), with grades 3 and 1, respectively. Of the three grade 3 events, two occurred on treatment and one occurred post-treatment. All grade 3 events were transient (duration: 1, 14 and 24 days). No events led to treatment modification.

The incidences of haematological and renal TEAEs were examined by number of prior treatments and prior treatment type, but no trends were identified. No clinically significant findings emerged from the monitoring of other lab parameters, as well as vital signs and ECGs of each participant during treatment and follow-up.

The toxicity profile during additional treatment was similar to the initial treatment. Renal TEAEs were observed in 8.3% (1/12) of additionally treated patients (renal failure). No additionally treated patient experienced grade ≥ 3 haematological or renal TEAEs.

## Discussion

The NETTER-R study presented the effects of ^177^Lu-DOTATATE treatment on PFS (median 24.8 months) and OS (median 41.4 months) in patients with panNETs, which is largely in line with previous clinical trials. In the phase I/II Erasmus MC study, the median PFS was 30.4 months. While comparing patient populations in the two trials, it was noted that patients in the Erasmus MC study often had a different tumour status to that of NETTER-R patients, had received fewer treatments prior to ^177^Lu-DOTATATE and fewer had progressive tumours at the time of therapy [18].

Disease progression recorded in approximately 30% of patients during the first 9 months of the study may have been due to the advanced disease stage of the participants. The median time from GEP-NET diagnosis was 3.5 years, and the median duration of metastatic disease in study participants was more than 3 years before starting ^177^Lu-DOTATATE treatment. Tumour grade may also have had an impact, as all G3 patients progressed within the first 9 months. In addition, this patient population was heavily pre-treated, with 28.2% of study participants having received ≥ 4 anticancer treatments prior to ^177^Lu-DOTATATE, and a portion of them continued to progress within the first months of the study.

Interestingly, the results of NETTER-R suggest that the type of treatment received prior to ^177^Lu-DOTATATE treatment may also affect the response to this agent. Survival analysed by multivariate modelling appeared longer in patients who had not received chemotherapy before enrolment in the NETTER-R study compared with those who had received at least one prior chemotherapy regimen. Patients who had received fewer treatments prior to ^177^Lu-DOTATATE appeared to demonstrate improved survival, which was expected as prior therapies are indicative of a more advanced disease. These results indicate a potential benefit of ^177^Lu-DOTATATE as an early treatment option. Its favourable tolerability suggests that it would not preclude other treatment options.

This study has shown that in a real-world population of patients with advanced panNETs, ^177^Lu-DOTATATE was well tolerated, with a safety profile consistent with the NETTER-1 and Erasmus MC trials [17–19]. Some TEAEs were observed, including haematological and renal toxicities, which were consistent with previous trials. Grade 3 anaemia, lymphopenia and thrombocytopenia occurred in one (0.9%), six (5.4%) and one (0.9%) patients, respectively, while renal TEAEs occurred in six patients. However, no TEAEs led to treatment discontinuation. MDS was an AE of potential interest with ^177^Lu-DOTATATE due to the long-term risk of patients developing MDS or AL following treatment [17–19]. In the NETTER-1 study, MDS was reported to occur in 1.8% (2/111) of patients with midgut NETs treated with ^177^Lu-DOTATATE [19]. In other studies, MDS was documented in 2.35% and 1.4% of patients treated with ^177^Lu-DOTATATE [20, 21]. In the present study of patients with panNETs, no MDS or AL was reported during follow up; however, it is worth noting that the median follow-up period (24.5 months [range: 2.0–123.4 months]) may have been insufficient time for these events to occur. In the Erasmus MC study, MDS and AL were observed to develop approximately 28 months (range: 9–41 months) and 55 months (range: 32–125 months) after the end of treatment, respectively [18]. In NETTER-1, MDS occurred earlier than 28 months (the median duration of follow-up was 14.0 months) [17].

The treatment administration pattern in this collection of real-world data showed that some patients received additional ^177^Lu-DOTATATE administrations after completion of initial treatment cycles. These additional treatment administrations were usually initiated at standard activity (7.4 GBq), were well tolerated and had a safety profile similar to that of the initial treatment.

The limitations of the present study are mainly related to the availability of certain data items, while not all variables contain the information in the same detail. This can be largely attributed to the fact that this was a non-interventional study with retrospective data collection from medical records, which was largely dependent on clinical practice and standard of care at each site included in the study. There was no uniformity in medical records across hospitals and regions, and the content of patient assessments during treatment and follow-up differed between sites. A total of 12 (10.9%) patients with non-functioning status were not assessed, and 2 (1.8%) patients with non-functioning status had missing information because tumour status was not available or not assessed by some institutions. In addition, there was no mandatory schedule of patient visits, so the intervals between evaluations or cycles of ^177^Lu-DOTATATE were inconsistent between patients. Due to the retrospective nature of the NETTER-R study, locally reviewed RECIST v1.1 data were collected according to local clinical practices and were not available for 48 (43.6%) patients. However, exploratory analyses showed that the availability of RECIST v1.1 evaluation data was not driven by demographic characteristics, histopathological profile or time from diagnosis. Another limitation is the chance of recall bias. Some of the subjects had started treatment several years before the start of the NETTER-R analysis, and safety data could not always be collected when patients were followed on a long-term in a different institution to the one where they received ^177^Lu-DOTATATE. Another limitation of the NETTER-R study may have been the selection of patients, which was off-label for 9 (8.2%) patients. While NETTER-1 selected patients based on the approved indication and did not include patients with grade 3 panNETs, NETTER-R included 3 (2.7%) patients with grade 3 tumours and 6 (5.5%) with missing information.

Overall, this retrospective real-world study conducted across sites in three countries supports recommendations in guidelines for ^177^Lu-DOTATATE use and reinforces the role of ^177^Lu-DOTATATE for the treatment of patients with SSTR-positive panNETs, a disease area with limited therapeutic options and an unmet need for novel treatments.

## Supplementary Information

Below is the link to the electronic supplementary material.

Supplementary file1 (DOCX 32 KB)

## Declarations

### Data collection

Data were collected either by an external researcher or a member of the patient’s care team, as required according to local regulations. Prespecified information was collected in the study-specific electronic case report forms.
