# Supplementary material for: Efficacy and safety of 177Lu‑DOTATATE in patients with advanced pancreatic neuroendocrine tumours: data from the NETTER-R international, retrospective study
Source: Eur J Nucl Med Mol Imaging. 2022 Apr 7;49(10):3529–37. doi: 10.1007/s00259-022-05771-3 (PMC9308585; doi:10.1007/s00259-022-05771-3)
Supplement: Supplementary file 1 — Supplementary file1 (DOCX 32 KB) [file 259_2022_5771_MOESM1_ESM.docx]

**SUPPLEMENTARY INFORMATION**

European Journal of Nuclear Medicine and Molecular Imaging

**Efficacy and safety of [^177^Lu]Lu‑DOTA-TATE in patients with advanced pancreatic neuroendocrine tumours: data from the NETTER-R international, retrospective study**

Dominique Clement, Shaunak Navalkissoor, Rajaventhan Srirajaskanthan, Frédéric Courbon, Lawrence Dierickx, Amy Eccles, Valerie Lewington, Mercedes Mitjavila, Juan Carlos Percovich, Benoît Lequoy, Beilei He, Ilya Folitar, John Ramage

**Corresponding author**

Dominique Clement

King’s College Hospital, London, UK

dominique.clement@nhs.net

**Supplementary Table 1** Baseline patient characteristics (SAS, *n*=110)

| **Patient demographics** |  |
| --- | --- |
| Age (years)  Mean (SD)  Q1–Q3  Median  Min–max  Weight (kg)  Mean (SD)  Q1–Q3  Median  Min–max  Sex, *n* (%)  Female  Male | 58 (12.2)  50.0–66.0  58.0  28–89  68.9 (15.8)  58.7–77.8  68.0  42.0–138.0  52 (47.3)  58 (52.7) |
| **Tumour evaluation** | ***n* (%)** |
| WHO NET grade | |
| NET, G1 | 30 (27.3) |
| NET, G2 | 71 (64.5) |
| NET, G3 | 3 (2.7) |
| Missing | 6 (5.5) |
| Ki-67 index^a^ | |
| ≤2% | 26 (23.6) |
| 3–20% | 73 (66.4) |
| >20% | 3 (2.7) |
| Missing | 8 (7.3) |
| Site of metastasis | |
| Liver | 105 (95.5) |
| Lymph nodes | 47 (42.7) |
| Bone | 32 (29.1) |
| Lungs | 4 (3.6) |
| Liver tumour burden | |
| Liver burden <10% | 9 (8.2) |
| Liver burden ≥10% to ≤25% | 32 (29.1) |
| Liver burden >25% or more than two liver lesions | 39 (35.5) |
| Missing | 30 (27.3) |
| Tumour status | |
| Functional | 33 (30.0) |
| Non-functional | 63 (57.3) |
| Not assessed | 12 (10.9) |
| Missing | 2 (1.8) |
| **Treatment history** | ***n* (%)** |
| Patients having previously received anticancer therapy | |
| Yes | 101 (91.8) |
| No | 9 (8.2) |
| Number of prior anticancer treatments | |
| 1 | 37 (33.6) |
| 2 | 24 (21.8) |
| 3 | 9 (8.2) |
| ≥4 | 31 (28.2) |
| Prior anticancer therapy | |
| ≥1 chemotherapy | 52 (47.3) |
| ≥1 protein kinase inhibitor^b^ | 42 (38.2) |
| ≥1 SSA | 77 (70.0) |
| ≥1 other anticancer therapy | 3 (2.7) |
| Progression at baseline^c^ | |
| Yes | 106 (96.4) |
| No | 4 (3.6) |

^a^Differences in Ki-67 index and WHO grade are due to the retrospective nature of the study and available data (some patients had missing Ki-67 index or WHO grade). ^b^Protein kinase inhibitors used included everolimus, sunitinib and dactolisib. ^c^Patients without progression at baseline were diagnosed with metastatic panNETs within 3 months of receiving the first cycle of [^177^Lu]Lu-DOTA-TATE

*G1* grade 1, *G2* grade 2, *G3* grade 3, *max* maximum, *min* minimum, *mTORi* mechanistic target of rapamycin inhibitor, *NET* neuroendocrine tumour, *panNET* pancreatic neuroendocrine tumour, *Q* quartile, *SAS* safety analysis set, *SD* standard deviation, *SSA* somatostatin analogue, *WHO* World Health Organization

**Supplementary Table 2** PRRT treatment characteristics

|  | **Initial treatment period *n*=110** | **Additional treatment period *n*=12** |
| --- | --- | --- |
| Cumulative activity (GBq), *n* (%) | | |
| <26.6 | 35 (31.8) | 11 (91.7) |
| 26.6–<32.6 | 72 (65.5) | 1 (8.3) |
| ≥32.6 | 3 (2.7) | 0 |
| Number of cycles by category, *n* (%) | | |
| 1 cycle | 7 (6.4) | 3 (25.0) |
| 2 cycles | 13 (11.8) | 7 (58.3) |
| 3 cycles | 12 (10.9) | 1 (8.3) |
| 4 cycles | 77 (70.0) | 1 (8.3) |
| 5 cycles | 1 (0.9) | 0 |
| Average activity per cycle (GBq) | | |
| Mean (SD) | 7.4 (0.58) | 6.9 (1.3) |
| Q1–Q3 | 7.4–7.6 | 7.1–7.6 |
| Median | 7.4 | 7.4 |
| Min–max | 3.7–8.3 | 3.7–7.9 |
| Missing | 0 | 0 |
| Number of cycles | | |
| Mean (SD) | 3.5 (1.0) | 2.0 (0.9) |
| Q1–Q3 | 3.0–4.0 | 1.5–2.0 |
| Median | 4.0 | 2.0 |
| Min–max | 1–5 | 1–4 |
| Missing | 0 | 0 |
| Total duration from first to last cycle (months)^a^ | | |
| Mean (SD) | 8.8 (4.2) | 6.4 (5.1) |
| Q1–Q3 | 7.0–10.6 | 3.2–6.9 |
| Median | 8.0 | 4.9 |
| Min–max | 2–30 | 2–19 |
| Missing | 0 | 0 |
| Average duration between treatment cycles per patient (weeks) | | |
| Median | 10.6 |  |
| Min–max | 8.4–15.0 |  |

^a^Duration of exposure to investigational drug (days) = (last date of exposure to [^177^Lu]Lu-DOTA-TATE) – (date of first administration of [^177^Lu]Lu-DOTA-TATE) + 1

*max* maximum, *min* minimum, *PRRT* peptide receptor radionuclide therapy, *Q* quartile, *SD* standard deviation

**Supplementary Table 3** Best tumour response

| **Tumour response^a^** | **RECIST v1.1, *n*=62** | **Investigator opinion 1, *n*=83** | **Investigator opinion 2, *n*=100** |
| --- | --- | --- | --- |
| ORR, *n* (%) | 25 (40.3) | 36 (43.4) | 54 (54.0) |
| CR | 0 | 0 | 2 (2.0) |
| PR | 25 (40.3) | 36 (43.4) | 52 (52.0) |
| SD | 22 (35.5) | 31 (37.3) | 34 (34.0) |
| PD | 13 (21.0) | 16 (19.3) | 12 (12.0) |
| NE | 2 (3.2) | 0 | 0 |

^a^Percentage is calculated relative to the number of patients with post-baseline tumour assessments

Investigator opinion 1 = RECIST v1.1 tumour assessments and radiological assessments. Investigator opinion 2 = all assessments available (radiological, clinical, metabolic and biomarker assessments)

*CR* complete response, *NE* not evaluable, *ORR* objective response rate, *PD* progressive disease, *PR* partial response, *RECIST v1.1* Response Evaluation Criteria in Solid Tumors version 1.1, *SD* stable disease

**Supplementary Table 4** PFS and OS by number of prior treatments

| **Number of prior treatments** | **RECIST v1.1**  **PFS, months (95% CI)** | **Investigator 1**  **PFS, months (95% CI)** | **Investigator 2**  **PFS, months (95% CI)** | **OS, months**  **(95% CI)** |
| --- | --- | --- | --- | --- |
| No prior treatment | 38.3 (1.4–72.2) (*n*=6) | 55.3 (1.4–72.2) (*n*=8) | 38.3 (1.4–72.2) (*n*=8) | NR (12.8–NE)  (*n*=9) |
| Any prior treatment | 23.5 (17.5–34.5) (*n*=56) | 23.5 (19.1–29.7) (*n*=75) | 23.3 (18.6–29.5) (*n*=92) | 41.4 (25.9–50.2) (*n*=101) |
| 1 | 34.5 (21.4–72.9) (*n*=17) | 29.7 (21.4–72.0) (*n*=25) | 34.5 (21.4–72.0) (*n*=34) | 73.6 (34.5–NE) (*n*=37) |
| 2 | 53.5 (9.0–61.5) (*n*=14) | 24.8 (19.8–53.5) (*n*=20) | 24.8 (10.9–53.5) (*n*=24) | 41.4 (20.0–NE) (*n*=24) |
| 3 | 19.1 (2.5–24.8) (*n*=7) | 15.3 (2.5–24.8) (*n*=8) | 14.8 (2.5–24.8) (*n*=8) | 23.3 (4.0–50.2) (*n*=9) |
| ≥4 | 13.1 (8.1–23.5) (*n*=18) | 14.0 (8.1–27.9) (*n*=22) | 12.7 (8.1–23.5) (*n*=26) | 21.5 (10.7–29.2) (*n*=31) |

*CI* confidence interval, *NE* not estimable, *NR* not reached, *OS* overall survival, *PFS* progression-free survival, *RECIST v1.1* Response Evaluation Criteria in Solid Tumors version 1.1

**Supplementary Table 5** TEAEs (SAS, *n*=110)

| **TEAEs** | ***n* (%)** |
| --- | --- |
| Subjects with at least one TEAE^a^ | 79 (71.8) |
| Subjects with at least one TEAE leading to study discontinuation | 0 |
| Subjects with treatment modification | 10 (9.1) |
| Subjects with at least one grade ≥3 TEAE | |
| Grade 3 | 25 (22.7) |
| Grade 4 | 5 (4.5) |
| Grade 5 | 3 (2.7) |
| Grade 5 TEAEs | |
| Abdominal abscess | 1 (0.9) |
| Metabolic encephalopathy | 1 (0.9) |
| Pulmonary embolism | 1 (0.9) |

^a^Defined as any AE starting after or on the day of the administration of [^177^Lu]Lu-DOTA-TATE. AEs are coded using MedDRA version 21.1. Toxicity grade: 3=severe, 4=threatening/disabling, 5=death
*AE* adverse event, *MedDRA* Medical Dictionary for Regulatory Activities, *SAS* safety analysis set, *TEAE* treatment-emergent adverse event

**Supplementary Table 6** Grade ≥3 haematological TEAEs (SAS, *n*=110)

| **Haematological toxicity** | **Anaemia,**  ***n* (%)** | **Lymphopenia, *n* (%)** | **Thrombocytopenia, *n* (%)** | **Neutropenia,**  ***n* (%)** |
| --- | --- | --- | --- | --- |
| Grade 3 | 1 (0.9) | 6 (5.4) | 1 (0.9) | 0 |
| Grade 4 | 0 | 0 | 0 | 0 |
| Grade 5 | 0 | 0 | 0 | 0 |

AEs are coded using MedDRA version 21.1. Toxicity grade: 3=severe, 4=threatening/disabling, 5=death
*AE* adverse event, *MedDRA* Medical Dictionary for Regulatory Activities, *SAS* safety analysis set, *TEAE* treatment-emergent adverse event
